# Surgical methods of total thyroidectomy for differentiated thyroid cancer: a systematic review and Bayesian network meta-analysis

**DOI:** 10.1097/JS9.0000000000000819

**Published:** 2023-11-02

**Authors:** Yuquan Yuan, Bin Pan, Enjie Tang, Hongbiao Mo, Junping Zhu, Ziying Yi, Dengwei Lu, Tingjie Yin, Yiceng Sun, Supeng Yin, Zeyu Yang, Fan Zhang

**Affiliations:** a Graduate School of Medicine, Chongqing Medical University; b Chongqing Institute Green and Intelligent Technology, Chinese Academy of Sciences; c Chongqing School, University of Chinese Academy of Sciences; d Department of Breast and Thyroid Surgery, Chongqing General Hospital; e Epidemiology Department, College of Preventive Medicine, Army Medical University (Third Military Medical University), Chongqing, People’s Republic of China

**Keywords:** differentiated thyroid cancer, thyroidectomy, meta-analysis, systematic review

## Abstract

**Background:**

Emerging remote-access surgical methods are utilized to treat differentiated thyroid cancer. The study aimed to compare the surgical integrity, safety, efficacy, and postoperative experience of patients among common surgical methods.

**Methods:**

The PubMed, Medline, Cochrane Library, Web of Science, and EMBASE databases were searched from their inception until March 2023. Pairwise meta-analysis and Bayesian network meta-analysis were performed. The surface under the cumulative ranking curve (SUCRA) was used to illuminate the probability that each method would be the best for each outcome.

**Results:**

Thirty-two studies comprising 7042 patients were included. Robotic bilateral axillo-breast approach (RBABA) and robotic gasless transaxillary approach (RGAA) retrieved fewer lymph nodes (LNs) than open thyroidectomy (OT). RBABA showed a significantly lower permanent recurrent laryngeal nerve (RLN) palsy rate than OT. According to SUCRA values, endoscopic transoral approach (EOA) ranked the highest in retrieved LNs (0.84), the proportion of stimulated serum thyroglobulin less than 1.0 ng/ml (0.77), and the pain score (0.77). Endoscopic bilateral areola approach (EBAA) ranked the highest in the transient RLN palsy rate (0.72). The endoscopic gasless transaxillary approach (EGAA) ranked the highest in the transient hypoparathyroidism rate (0.78). RBABA ranked the highest in the rate of permanent RLN palsy (0.94) and hypoparathyroidism (0.77). OT ranked the highest in operative time (0.92).

**Conclusions:**

Each surgical method of total thyroidectomy has benefits and limitations. EOA performed the best in maintaining surgical integrality and reducing the pain score, while taking a long operative time. Generally, RBABA showed the best advantage in protecting parathyroid glands and RLN but with the longest operative time. OT had the best advantage in operative time. Therefore, OT and EOA are ideal methods for patients with a higher risk of central LN metastasis. RBABA and EOA may not be suitable for elderly patients or those with high anesthesia risk.

## Introduction

Highlights

- The endoscopic transoral approach showed the best advantage in surgical integrality.
- The endoscopic transoral approach had the best advantage in pain score.
- The robotic bilateral axillo-breast approach ranked the highest in surgical safety.
- The robotic bilateral axillo-breast approach had the longest operative time.
- An open thyroidectomy had the shortest operative time.

Thyroid cancer is the most pervasive endocrine neoplasm, with 586 000 newly diagnosed cases worldwide, and it ranks in ninth place for incidence in 2020^1,2^. Thyroidectomy is the principal therapeutic strategy for differentiated thyroid cancer (DTC)^3^. The large neck incision and the occurrence of surgical injuries are major concerns of conventional open thyroidectomy (OT), leading to a call to action for surgical methods with excellent cosmetic outcomes. Therefore, remote-access thyroid surgery, including endoscopic thyroidectomy (ET) and robotic thyroidectomy (RT), have been developed^4^.

ET was first performed by Hüscher *et al*.^5^ in 1997 with advantages in minimizing invasive interventions and providing patients with esthetically pleasing results. Subsequently, various approaches to ET have been developed with benefits and limitations. For instance, the minimally invasive video-assisted approach (MIVAA), providing a relatively clear surgical field by magnification and lighting, also leaves a cervical scar at a length of ~2 cm^6,7^. The endoscopic transoral approach (EOA), characterized by eliminating surgical skin scars, is yet to be widely accepted due to the high risk of oral microbes related to infection^8,9^. The endoscopic bilateral areola approach (EBAA), with the advantage of dissecting the superior gland pole and the external branch of the superior laryngeal nerve, poses difficulties in central neck dissection because of the obstruction of the clavicle and sternum^10^. The endoscopic gasless transaxillary approach (EGAA), providing a wide surgical view from the lateral perspective, presents challenges in obtaining a view of the contralateral surgical field^11–13^. The endoscopic bilateral axillo-breast approach (EBABA) can provide bilateral visualization of major structures but with greater invasiveness, as it requires four incisions^12,14^.

Undoubtedly, ET and RT in the same approach have similar benefits and limitations but still exist specific differences. RT can provide a superior 3-dimensional magnified view, making it easier to identify main structures, such as vessels and recurrent laryngeal nerve (RLN)^4,15^. Furthermore, RT has the advantages of fine motion scaling, hand-tremor filtering and innovative instrumentation with extended freedom of motion^4^. Nevertheless, RT lacks the tactile sensation of a surgeon and costs more^16^. Although ET and RT have been demonstrated to have many advantages, there remain concerns about their safety and integrity^17^.

To comprehensively compare the surgical integrality, safety, efficiency, and postoperative experience of patients among different methods of total thyroidectomy, we conducted a network meta-analysis (NMA). We also ranked all surgical methods in different indexes to clarify the benefits and limitations of each surgical method, which was clinically meaningful. Furthermore, data from a regional academic medical center were included to further enhance the reliability of our results.

## Materials and methods

### Date collecting

This retrospective study was approved by the ethics committee of our center, which waived the requirement to obtain informed consent (approval number: KY 2021-040-01). The cohort comprised 112 patients diagnosed with DTC who underwent total thyroidectomy and central lymph node dissection and was stratified into two groups based on the surgical method: the EBAA group (*n*=58) and the OT group (*n*=54). Patients with prior neck surgery, potential invasion of the RLN, or suspicious lateral lymph node metastasis were excluded. All operations were performed by one experienced surgeon who has 20 years of surgical experience and performs at least 600 successful cases per year. Clinical data were extracted from medical records and compiled into a database, including patient age, sex, tumor size, operative time, number of retrieved lymph nodes (LNs), and complications (transient/permanent RLN palsy and hypoparathyroidism) (shown in Supplementary Table 1, Supplemental Digital Content 9, http://links.lww.com/JS9/B264).

### Meta-analysis

This NMA was conformed with PRISMA (Preferred Reporting Items for Systematic Reviews and Meta-Analyses) and AMSTAR (Assessing the methodological quality of systematic reviews) Guidelines^18,19^.

### Literature search strategy

We performed a systematic literature search of PubMed, Medline, Cochrane Library databases, Web of Science, and EMBASE published up to March 2023 to identify all relevant studies. The keywords were (ʻDifferentiated thyroid cancerʼ OR ʻThyroid malignancyʼ OR ʻThyroid carcinomaʼ OR ʻThyroid cancerʼ) AND (ʻThyroidectomyʼ OR ʻConventional thyroidectomyʼ OR ʻOpen thyroidectomyʼ) OR (ʻEndoscopy thyroidectomyʼ OR ʻVideo-assisted surgeryʼ OR ʻLaparoscopyʼ) OR (ʻRobotic thyroidectomyʼ OR ʻDa Vinci surgical systemʼ OR ʻRobot-Assisted Surgeryʼ). In addition, reference lists of the retrieved articles were reviewed to obtain other eligible studies.

Two reviewers independently conducted the literature search, and disagreements were solved by consensus. The abstracts of the retrieved studies were reviewed and excluded if deemed irrelevant. The full-text was checked to determine the final eligible articles. Discrepancies were resolved by discussion with a third reviewer.

### Inclusion and exclusion criteria

Studies were selected according to the following inclusion criteria: (1) Study design: observational study. (2) Population: patients with DTC who have undergone total thyroidectomy. (3) Intervention: studies comparing at least two surgical methods, including ET, OT, and RT. (4) Outcomes: studies reporting at least one outcome of interest mentioned below. (5) Language: studies that were published in English. The exclusion criteria were as follows: (1) Case reports, reviews, letters to the editor, etc. (2) Unilateral thyroidectomy or subtotal thyroidectomy. (3) Other types of thyroid cancer. (4) Duplicate articles.

### Data extraction and quality evaluation

The methodological quality of the observational research was assessed by the Newcastle–Ottawa Scale (NOS)^20^. Three broad subscales included the study group selection (0–4 points), group comparability (0–2 points), and exposure and outcome elucidation (0–3 points). A score of 4–6 was defined as moderate, and a score of 7 or more was considered high-quality. The same two independent reviewers independently reviewed the full-text, quality assessment, and data extraction. If discrepancies arose, a consensus was reached by consulting the third reviewer and comprehensively comparing the data. The extracted data mainly contained the following: (1) Study information: first author, publication years, countries, and study design. (2) Baseline characteristics: age, sex, tumor size, pathology, surgical methods, and surgical approaches. (3) Outcome information: 1). Surgical integrity: number of retrieved LNs and the proportion of thyroid-stimulating hormone-stimulated serum thyroglobulin (STG) less than 1.0 ng/ml. 2). Surgical safety: transient/permanent RLN palsy rate and transient/permanent hypoparathyroidism rate. 3). Surgical efficiency: operative time, volume of drainage, and postoperative hospital stay. 4). postoperative experience of patients: pain score.

### Statistical analysis

First, we performed a pairwise meta-analysis (PMA) for direct comparisons reported at least twice by STATA statistical software (version 14.0; StataCorp)^21^. Continuous data were analyzed using the mean difference (MD) with a corresponding 95% CI, and dichotomous data were measured using the odds ratio (OR) with a corresponding 95% CI. Heterogeneity tests were performed based on the *Q* test and *I*^2^ statistics. The heterogeneity of the effect size across the studies was tested using the Q statistic (*P*<0.05 was considered heterogeneous) and the *I*^2^ statistic (*I*^2^ >50% was considered heterogeneous). If there was significant heterogeneity between studies, a random-effects model was used; otherwise, a fixed-effects model was used.

Bayesian NMA was performed using Markov Chain Monte Carlo (MCMC) methods in JAGS version 4.3 to allow indirect comparisons among treatment interventions. The resultant effects were reported as posterior median MD or I with the corresponding 95% CI. The analysis was performed using 1000 burn-ins, 50 000 iterations, and 20 000 adaptations, after which the model fit was tested using a diagram of leverage. The model fit between random and fixed effect models was compared according to the deviance information criterion (DIC), and considering a lower DIC implied a better model^22^. The loop-specific approach was used to evaluate the agreement between direct and indirect effects in all closed loops^23–25^. A *P*-value ≥0.05 suggested that the consistency of the model was satisfactory. Heterogeneity was evaluated and reported as tau (τ). A multivariate meta-regression analysis was performed to identify factors with a nonnegligible effect when the τ value >0.5. To rank treatments for each outcome, we used the surface under the cumulative ranking curve (SUCRA) and the mean ranks. A sensitivity analysis was conducted through the sequential exclusion of each incorporated study to observe whether there were significant changes in the combined results. Publication and reporting bias were estimated by adjusted funnel plots and the Egger test. The statistical analyses were conducted in R 3.6.2 and STATA (version 14.0).

## Results

### Literature search

Following the previous search strategy, a total of 1574 potentially relevant articles were identified, and 56 remained after duplicate removal and initial screening. Then, a full-text review was conducted to exclude articles not meeting the inclusion criteria, and 31 studies were identified. Finally, combined with our data, 32 studies with 7042 patients were enrolled in this meta-analysis^13,16,26–54^. The PRISMA flow diagram shows the article selection and exclusion procedure details (Supplementary Figure 1, Supplemental Digital Content 1, http://links.lww.com/JS9/B255).

### Study characteristics

The basic characteristics of the included studies are presented in Table 1. The mean age of the patients ranged from 30.8 to 51.8 years. Of the 32 publications, 14 were retrospective studies, 9 were propensity score-matched retrospective studies, nine were prospective studies. The study period ranged from January 2000 to May 2022. The meta-analysis included eight surgical methods: EBAA, EBABA, EGAA, EOA, MIVAA, OT, the robotic bilateral axillo-breast approach (RBABA), and the robotic gasless transaxillary approach (RGAA). Most of the studies (29/32, 91%) were performed in the Asia-Pacific region (China, Korea, and Thailand).

**Table 1:** Characteristics of the 32 included studies.

Study	Year	Country	Method	Age (mean)	Sex (male/female)	Sample size	Average tumor size (cm)	Design	NOS	REF
Lee *et al*.	2010	Korea	RGAA	NA	NA	26	NA	Retro	6	^26^
			OT	NA	NA	26	NA			
Kim *et al*.	2011	Korea	RBABA	41.3	6/63	69	0.6	Retro	7	^27^
			EBABA	39.9	2/93	95	0.6			
			OT	51.8	34/104	138	0.7			
Lee *et al*.	2011	Korea	RBABA	43.7	17/91	108	0.8	Retro Ma	8	^28^
			OT	43.8	17/91	108	0.8			
Lee *et al*.	2012	Korea	RGAA	NA	NA	27	NA	Retro	7	^29^
			OT	NA	NA	90	NA			
Lombardi *et al*.	2012	Italy	MIVAA	37.3	3/49	52	1.2	Retro Ma	8	^30^
			OT	38	10/42	52	1.3			
Noureldine *et al*.	2013	America	RGAA	NA	NA	14	NA	Retro	7	^31^
			OT	NA	NA	25	NA			
Ryu *et al*.	2013	Korea	RGAA	39	3/42	45	1	Pro	8	^32^
			OT	48.9	9/36	45	1.2			
Yi *et al*.	2013	Korea	RGAA	42.1	0/98	98	NA	Retro	7	^33^
			OT	51.8	0/423	423	NA			
Lee *et al*.	2014	Korea	RGAA	40.5	0/60	60	1	Pro	7	^34^
			OT	45.5	0/56	56	1			
Kim *et al*.	2014	Korea	RBABA	NA	NA	100	NA	Retro Ma	6	^35^
			OT	NA	NA	108	NA			
Lee *et al*.	2014	Korea	RGAA	39.8	NA	43	1.1	Pro	7	^36^
			OT	48.3	NA	51	1.1			
Chai *et al*.	2016	Korea	RBABA	36	0/27	27	0.8	Pro	8	^37^
			OT	38.9	0/27	27	0.9			
He *et al*.	2016	China	RBABA	40.9	9/41	50	0.5	Pro	8	^38^
			OT	41.5	8/42	50	0.5			
Hensler *et al*.	2016	America	MIVAA	46	6/43	49	NA	Retro	7	^39^
			OT	48.6	12/26	38	NA			
Huang *et al*.	2016	China	EGAA	37.8	16/59	75	0.5	Pro	8	^40^
			OT	39.2	31/92	123	0.5			
Kim *et al*.	2016	Korea	EBABA	NA	NA	56	NA	Retro	7	^41^
			OT	NA	NA	684	NA			
Kim *et al*.	2016	Korea	RBABA	38.9	6/106	112	0.5	Retro	8	^42^
			OT	50.4	11/106	117	1			
Koh *et al*.	2016	Korea	EGAA	NA	NA	43	NA	Retro	6	^43^
			OT	NA	NA	635	NA			
Lee *et al*.	2016	Korea	RGAA	41.2	24/182	206	1.1	Retro Ma	8	^44^
			OT	42.1	25/181	206	1.1			
Park *et al*.	2016	Korea	EGAA	38	4/46	50	0.8	Pro	7	^45^
			OT	50.8	14/88	102	0.8			
Xiang *et al*.	2016	China	EBAA	34.2	0/49	49	0.8	Retro	7	^46^
			OT	46.9	6/41	47	1.2			
Chai *et al*.	2017	Korea	RBABA	30.8	2/19	21	2.8	Retro	8	^47^
			OT	51.6	13/52	65	2.8			
Kim *et al*.	2017	Korea	RBABA	NA	NA	114	NA	Retro Ma	7	^16^
			EBABA	NA	NA	114	NA			
Kim *et al*.	2017	Korea	EGAA	39.5	8/192	200	1	Retro	7	^13^
			OT	48.9	138/400	538	0.9			
Anuwong *et al*.	2018	Thailand	EOA	NA	NA	86	NA	Retro Ma	9	^48^
			OT	NA	NA	84	NA			
Paek *et al*.	2018	Korea	RBABA	36.4	5/66	71	0.8	Retro	7	^49^
			OT	47.2	74/231	305	0.8			
Bae *et al*.	2019	Korea	RBABA	40.8	7/116	123	0.8	Retro Ma	8	^50^
			OT	41.1	14/232	246	0.8			
Zhang *et al*.	2019	China	EBAA	NA	NA	50	NA	Pro	7	^51^
			OT	NA	NA	50	NA			
Ahn *et al*.	2020	Korea	EOA	NA	NA	40	NA	Pro	6	^52^
			OT	NA	NA	85	NA			
Hong *et al*.	2020	Korea	EOA	NA	NA	12	NA	Retro Ma	7	^53^
			OT	NA	NA	13	NA			
Paek *et al*.	2022	Korea	RBABA	NA	NA	54	NA	Retro Ma	7	^54^
			OT	NA	NA	54	NA			
Yuan *et al*.	2023	China	EBAA	34.7	3/55	58	0.7	Retro	8	Present study
			OT	34.9	7/47	54	0.9			

EBAA, endoscopic bilateral areola approach; EBABA, endoscopic bilateral axillo-breast approach; EGAA, endoscopic gasless transaxillary approach; EOA, endoscopic transoral approach; MIVAA, minimally invasive video-assisted approach; NA, not available; NOS, the score of included study assessed by the Newcastle–Ottawa Scale; OT, open thyroidectomy; Pro, prospective study; RBABA, robotic bilateral axillo-breast approach; RGAA, robotic gasless transaxillary approach; RER, references; Retro, retrospective study; Retro Ma, propensity score-matched retrospective study.

The overall quality assessment of the 32 studies was assessed by the NOS score. All studies were regarded as high-quality, with a score of six or higher. Detailed information on the quality evaluation is displayed in Supplementary Table 2. The network relationships between different surgical methods are shown in Supplementary Figure 2 (Supplemental Digital Content 2, http://links.lww.com/JS9/B256). The size of each circle illustrates the number of included patients, and the thickness of the lines between the two surgical methods reflects the number of studies. After 50 000 iterations, the model achieved ideal convergence (Supplementary Figure 3, Supplemental Digital Content 3, http://links.lww.com/JS9/B258). The cumulative ranking plots and rank nomograms of the eight surgical methods are shown in Supplementary Figure 4 (Supplemental Digital Content 4, http://links.lww.com/JS9/B259). The risk of bias is presented in Supplementary Figure 5 (Supplemental Digital Content 5, http://links.lww.com/JS9/B260).

## Meta-analysis

### Surgical integrity

**Figure 1:**
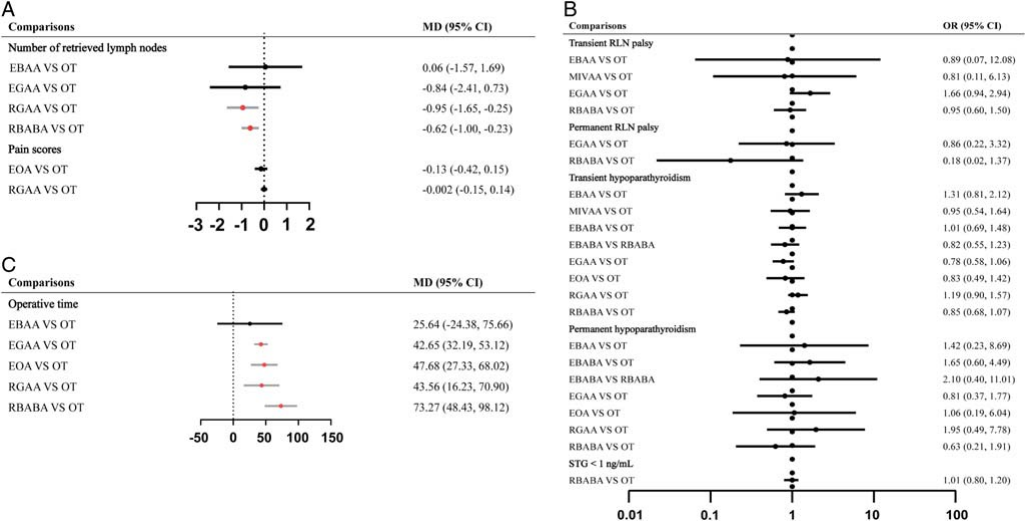
Forest plot comparison of the different surgical methods for all outcomes. EBAA, endoscopic bilateral areola approach; EBABA, endoscopic bilateral axillo-breast approach; EGAA, endoscopic gasless transaxillary approach; EOA, endoscopic transoral approach; MD, mean difference; MIVAA, minimally invasive video-assisted approach; OT, open thyroidectomy; OR, odds ratio; RBABA, robotic bilateral axillo-breast approach; RGAA, robotic gasless transaxillary approach; RLN, recurrent laryngeal nerve.

**Figure 2:**
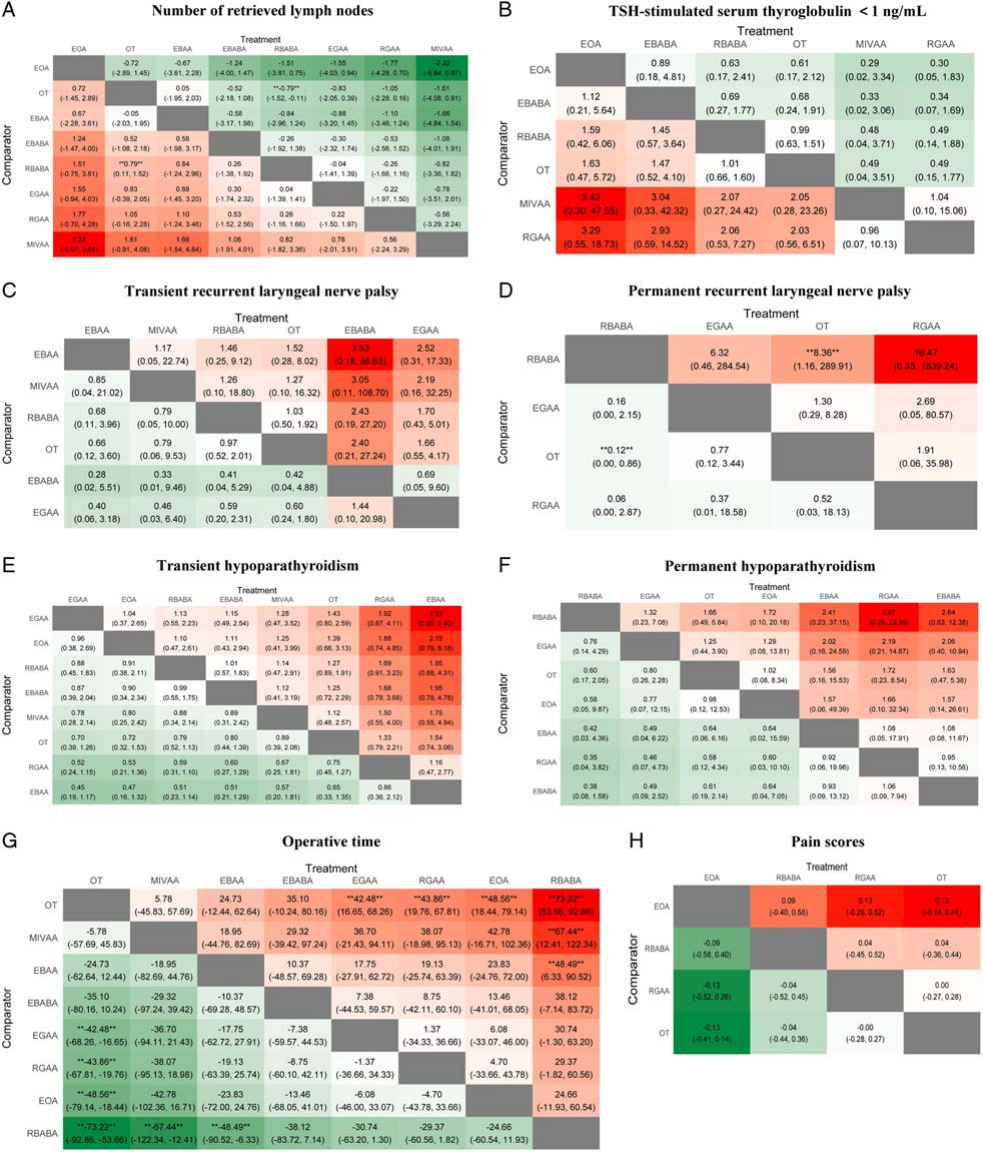
Heat plots of the league table for the eight surgical methods. EBAA, endoscopic bilateral areola approach; EBABA, endoscopic bilateral axillo-breast approach; EGAA, endoscopic gasless transaxillary approach; EOA, endoscopic transoral approach; MIVAA, minimally invasive video-assisted approach; OT, open thyroidectomy; RBABA, robotic bilateral axillo-breast approach; RGAA, robotic gasless transaxillary approach.

**Figure 3:**
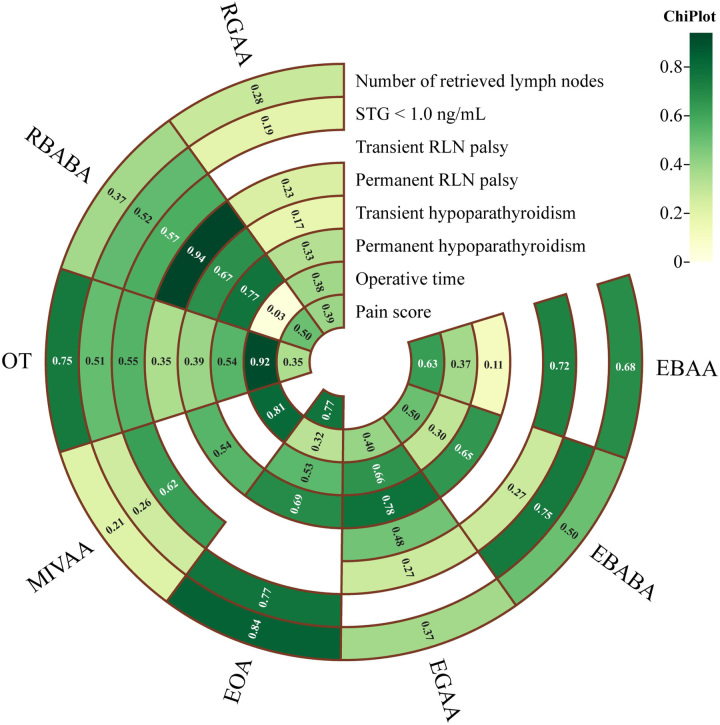
The surface under the cumulative ranking curve values of all outcomes for eight surgical methods. The darker color, consistent with the higher SUCRA value, indicates greater superiority. SUCRA, surface under the cumulative ranking curve; STG, TSH-stimulated serum thyroglobulin; RLN, recurrent laryngeal nerve.

RBABA retrieved fewer LNs than OT as assessed by PMA and NMA (Fig. 1A, Fig. 2A). PMA indicated that RGAA retrieved fewer LNs than OT (MD: −0.946, 95% CI: −1.645–0.247, *P*=0.008) (Fig. 1A). PMA and NMA revealed no significant differences in the proportion of STG less than 1.0 ng/ml among EBABA, EOA, MIVAA, OT, RBABA, and RGAA (Fig. 1B, Fig. 2B). Notably, EOA had the highest SUCRA value for both the number of retrieved LNs (0.84) and the proportion of STG less than 1.0 ng/ml (0.77) (Fig. 3), suggesting its superiority in surgical integrity. More results involving the SUCRA values of other surgical methods for surgical integrity are shown in Figure 3 and Supplementary Table 3 (Supplemental Digital Content 9, http://links.lww.com/JS9/B264).

### Surgical safety

PMA and NMA indicated no differences in transient RLN palsy or transient/permanent hypoparathyroidism among all surgical methods (Fig. 1B, Fig. 2C, E, F). RBABA showed a significantly lower permanent RLN palsy rate than OT (OR: 0.12, 95% CI: 0.00–0.86) in the NMA (Fig. 2D). Interestingly, RBABA demonstrated the best advantages in reducing the incidence of permanent RLN palsy and hypoparathyroidism, with the highest SUCRA values of 0.94 and 0.77, respectively (Fig. 3). More results regarding the SUCRA values of other surgical methods for surgical safety are available in Figure 3 and Supplementary Table 3 (Supplemental Digital Content 9, http://links.lww.com/JS9/B264).

### Surgical efficiency

Significant differences in operative time were observed among different surgical methods. PMA and NMA showed that EGAA, EOA, RBABA, and RGAA had longer operative times than OT (Fig. 1C, Fig. 2G). Concomitantly, the operative time of RBABA was relatively longer than that of MIVAA (MD: 67.44, 95% CI: 12.41–122.34) and EBAA (MD: 48.49, 95% CI: 6.33–90.52) according to NMA (Fig. 2G). Generally, OT effectively shortened the operative time, outperforming all other surgical methods, with the highest SUCRA value of 0.92 (Fig. 3 and Supplementary Table 3, Supplemental Digital Content 9, http://links.lww.com/JS9/B264). There were no significant differences among different surgical methods in the volume of drainage and postoperative hospital stay (Supplementary Figure 6, Supplemental Digital Content 6, http://links.lww.com/JS9/B261, 7, Supplemental Digital Content 7, http://links.lww.com/JS9/B262).

### Postoperative experience of patients

PMA and NMA showed no significant differences in pain scores among the EOA, OT, RBABA, and RGAA groups (Fig. 1A, Fig. 2H). According to the SUCRA values for pain scores, EOA (0.77) ranked as the best surgical approach, followed by RBABA (0.50), RGAA (0.39), and OT (0.35) (Fig. 3 and Supplementary Table 3, Supplemental Digital Content 9, http://links.lww.com/JS9/B264).

**Figure 4:**
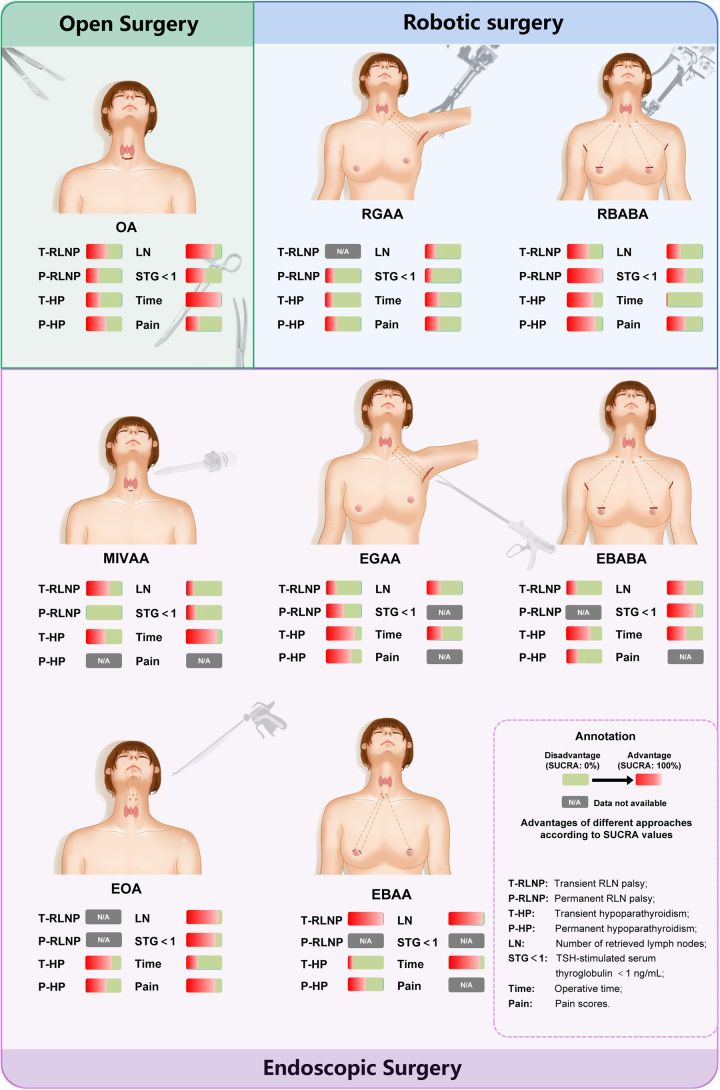
Comparisons of the different surgical methods. The comparisons of the different surgical methods are based on SUCRA values. A greater proportion of red color indicates higher superiority. EBAA, endoscopic bilateral areola approach; EBABA, endoscopic bilateral axillo-breast approach; EGAA, endoscopic gasless transaxillary approach; EOA, endoscopic transoral approach; MIVAA, minimally invasive video-assisted approach; OT, open thyroidectomy; RBABA, robotic bilateral axillo-breast approach; RGAA, robotic gasless transaxillary approach; SUCRA, surface under the cumulative ranking curve.

Overall, these results suggested that different surgical methods had unique advantages. For this reason, we compared these surgical methods according to their SUCRA values and summarized our findings in Fig. 4.

### Inconsistency, heterogeneity, sensitivity analysis, and publication bias

Inconsistency and heterogeneity are shown in Supplementary Table 4 (Supplemental Digital Content 9, http://links.lww.com/JS9/B264). No significant local inconsistency was found within the meta-analysis of eight surgical methods. Heterogeneity was low (τ, <0.10) for the proportion of STG less than 1.0 ng/ml, transient RLN palsy, transient hypoparathyroidism, and permanent hypoparathyroidism. Overall, the heterogeneity was reasonable for the number of retrieved LNs in eight methods (τ, 0.35). In contrast, the heterogeneity was higher for the operative time (τ, >1.00). A sensitivity analysis was conducted by deleting individual studies and replacing the effect models. However, the overall statistical significance did not change, suggesting that the results were robust and reliable. No ʻsmall-studyʼ effect was present (Supplementary Figure 5, Supplemental Digital Content 5, http://links.lww.com/JS9/B260) by visual evaluation except for operative time. Notably, the Egger test showed no significant *P*-values for operative time (*P*=0.89) (shown in Supplementary Figure 8, Supplemental Digital Content 8, http://links.lww.com/JS9/B263).

### Meta-regression analysis

Meta-regression was performed for the operative time (Supplementary Table 5, Supplemental Digital Content 9, http://links.lww.com/JS9/B264). Analysis of operative time did not show significant influences in publication years, publication types, or countries.

## Discussion

This is the first NMA to compare the surgical integrality, safety, efficiency, and postoperative experience of patients among eight surgical methods of total thyroidectomy. As the total thyroidectomy was the major concern of this study, cohorts associated with unilateral lobectomy and subtotal thyroidectomy were excluded. Considering that the implementation standards of surgical procedures in different countries or regions might influence the conclusions of this paper, we thoroughly reviewed the methods section of each original study, classifying them rigorously according to their surgical procedures. Next, propensity score-matched cohorts were selected to reduce selective bias. The results confirmed that the surgical outcomes of remote-access thyroidectomy were comparable to OT, even superior to OT in some outcomes. Additionally, we also demonstrated the benefits and limitations of each surgical method.

Regarding surgical integrity, consistent with previous studies^55–58^, we found that there were no significant differences among all surgical methods except that RGAA and RBABA retrieved fewer LNs compared to OT. According to the SUCRA values, RBABA outperformed RGAA, potentially because RBABA could obtain bilateral surgical views more easily than RGAA^11,12,17,59^. Moreover, EOA had the best advantage in retrieving LNs among all surgical methods, which may relate to the central compartment being easy to access under EOA^60,61^. The proportion of STG less than 1.0 ng/ml is a critical indicator of surgical integrity in total thyroidectomy^28^. Our results showed there were no statistical differences among all surgical methods. EOA had the best advantage in the proportion of STG less than 1.0 ng/ml (SUCRA, 0.77), which was consistent with its superiority in retrieving LNs. Overall, surgeons need to pay more attention to the resection of LNs when performing RT. EOA and OT are recommended for the patient with a high risk of LN metastasis.

Protecting the parathyroid glands (PGs) and nerves is a topic of constant attention in thyroid surgery. Our results demonstrated there were no statistical differences in surgical complications among all surgical methods except that RBABA had a significantly lower incidence of permanent RLN injury than OT. This may relate to the preferable 3-dimensional magnified view of RT and the relatively clearer bilateral surgical view^28,59^. According to SUCRA values, EGAA had the best advantage in transient hypoparathyroidism, while had the least advantage in transient RLN palsy. Because the gasless transaxillary approach provides a wide surgical view to identify the PGs and RLN clearly from the lateral perspective, but has challenges in obtaining a view of the contralateral surgical field, especially for the RLN in the contralateral surgical field^4,11,17^. Interestingly, RBABA was superior to OT in protecting PGs and RLN, while RGAA was inferior to OT in these aspects. Other complications, such as seroma, wound infection, and postoperative bleeding, failed to be analyzed due to data scarcity, which was a limitation of the present study. Overall, RBABA may protect PGs and RLN better than other surgical methods.

Regarding surgical efficiency, RBABA had the longest operative time among all surgical methods, possibly relating to the time spent on four surgical incisions and robotic arm docking^4,16^. It is widely accepted that ET has a shorter operative time than RT, while according to the results of SUCRA values, EOA had a longer operative time than RGAA. This may associate with the long learning curve of EOA^48,62^. Although RBABA had the advantage of controlling complications and EOA had benefits in terms of surgical integrality, in our viewpoint, neither RBABA nor EOA was recommended for elderly patients because of a high anesthesia risk and prolonged operative time. In addition, by analyzing the volume of drainage and postoperative hospital stay, we found that there were no significant differences among different surgical methods. Undoubtedly, these conclusions require further demonstration, because both the volume of drainage and postoperative hospital stay are closely related to patient management patterns in different clinical centers.

Pain scores and cosmetic outcomes are primary aspects of the postoperative experience of patients. Undoubtedly, ET and RT exhibit excellent cosmetic performance compared to OT^17,63^. Therefore, assessing the pain scores among different methods is meaningful. Similar to previous studies^58^, our results showed no significant differences in pain scores among all surgical methods. However, according to SUCRA values, EOA had the best advantage in pain score, possibly because EOA had less flap dissection than other approaches^61,64^. Although remote-access thyroidectomy requires flap dissection, they have potential advantages in relieving postoperative pain.

In this study, total thyroidectomy through different approaches was compared cross-sectionally in terms of safety, integrity, efficiency, and the postoperative experience of patients, which revealed their benefits and limitation. However, as most previous original studies used OT as the control group to discuss the advantages and disadvantages of different remote-access surgical methods, the direct pairwise comparisons between them were limited. In this NMA, OT was employed as the ʻintermediateʼ to enable indirect comparisons between different remote-access surgical methods, which resulted in more than half of the patients included in this study undergoing OT. Nevertheless, we still anticipate more large, multicenter studies in the future to provide broader direct comparisons between the different surgical methods. Unfortunately, several remote-access surgical methods were not involved in this study. The retroauricular thyroidectomy was first described by Schardey *et al*.^65^ in 2008 with a smaller dissection area to reach the thyroid gland. It is a cosmetic and feasible surgical approach, as the scar is concealed by the auricle and hair^66^. Additionally, it has a small dissection area to reach the thyroid gland^17,65^. However, we did not include it in this study because most previous studies pertained to unilateral retroauricular thyroidectomy^67–69^.

This study has several limitations. As many studies failed to provide the outcomes of unilateral thyroidectomy and total thyroidectomy separately, the number of included studies was limited. Additionally, many of the included investigations were retrospective studies from East Asia that may have caused potential selection and reporting biases, yet we tried to select propensity core-matched cohorts to reduce such selective bias. Moreover, due to the scarcity of collected data, the recurrence rates and other surgical complications, such as seroma, wound infection, and postoperative bleeding, were not analyzed in this meta-analysis. Finally, the primary focus of this study was to investigate the benefits of different surgical methods on patient populations. However, the individual condition of different patients can have a certain impact on surgical outcomes. Given the potential confounding factors, most of literatures included in our study design are randomized controlled trials or data adjusted using propensity scores. It can largely reduce the influence of individual patient variability, ensuring the quality of our findings.

## Conclusion

Benefits and limitations are obvious to each surgical method of total thyroidectomy according to SUCRA values. EOA performed the best in maintaining surgical integrality and reducing the pain score, while taking a long operative time. Generally, RBABA showed the best advantage in protecting PGs and RLN but with the longest operative time. OT had the best advantage in operative time. Therefore, OT and EOA are ideal methods for patients with a higher risk of central LN metastasis. RBABA and EOA may not be suitable for elderly patients or those with high anesthesia risk. A comprehensive surgical consideration should base on the benefits and limitations of different surgical methods, and conditions of the patient.

## Ethical approval

This study was approved by The Ethical Committee of Chongqing General Hospital (approval number: KY 2021-040-01).

## Consent

This study was approved by The Ethical Committee of Chongqing General Hospital. As a retrospective study, the requirement to obtain informed consent was waived.

## Sources of funding

This work was supported by the Key Special Project for Technological Innovation and Application Development of Chongqing (Grant No. CSTB202TIAD-KPX0177), the Chongqing Medical Scientific Research Project (Joint Project of Chongqing Health Commission and Science and Technology Bureau, Grant No. 2023QNXM017), and the Basic Research and Frontier Exploration Project of Yuzhong District, Chongqing, China (Grant No. 20210162).

## Author contribution

MSc Yuan and MSc Pan take responsibility for the integrity of the data and the accuracy of the data analysis. MSc Yuan and MSc Pan contributed equally to this study and share first authorship. Y. and P.: conceptualization, data curation, formal analysis, methodology, writing – original draft; T., M., Z., Y., L., Y., and S.: data curation, investigation, and funding acquisition; Y., Y., and Z.: funding acquisition, supervision, and writing – review and editing.

## Conflicts of interest disclosure

No conflict of interest exists in the submission of this manuscript, and the manuscript is approved by all authors for publication.

## Research registration unique identifying number (UIN)

1. Name of the registry: not applicable.
2. Unique identifying number or registration ID: not applicable.
3. Hyperlink to your specific registration (must be publicly accessible and will be checked): not applicable.

## Guarantor

Supeng Yin, MD, PhD, e-mail: yinsupeng@163.com, Zeyu Yang, MD, e-mail: yangzeyucgh@163.com, Fan Zhang, MD, PhD, e-mail: zhangfancgh@163.com.

## Data availability statement

The raw data were all collected in the included studies. Unavailable data were extracted in graph by statistic software or from authors that were contacted in attempt to reach the original data. We declare the authenticity of the data.

## Provenance and peer review

Not commissioned, externally peer-reviewed.

## Supplementary Material
