# Supplementary material for: Surgical methods of total thyroidectomy for differentiated thyroid cancer: a systematic review and Bayesian network meta-analysis
Source: Int J Surg. 2023 Nov 2;110(1):529–40. doi: 10.1097/JS9.0000000000000819 (PMC10793844; doi:10.1097/JS9.0000000000000819)
Supplement: Supplementary file 9 [file js9-110-529-s009.pdf]

Surgical methods of total thyroidectomy for differentiated thyroid cancer: A systematic review and Bayesian network meta-analysis

Yuquan Yuan, MSc[a,b,c,d,1]; Bin Pan, MSc[a,b,c,d,1]

Supplementary Figure 1

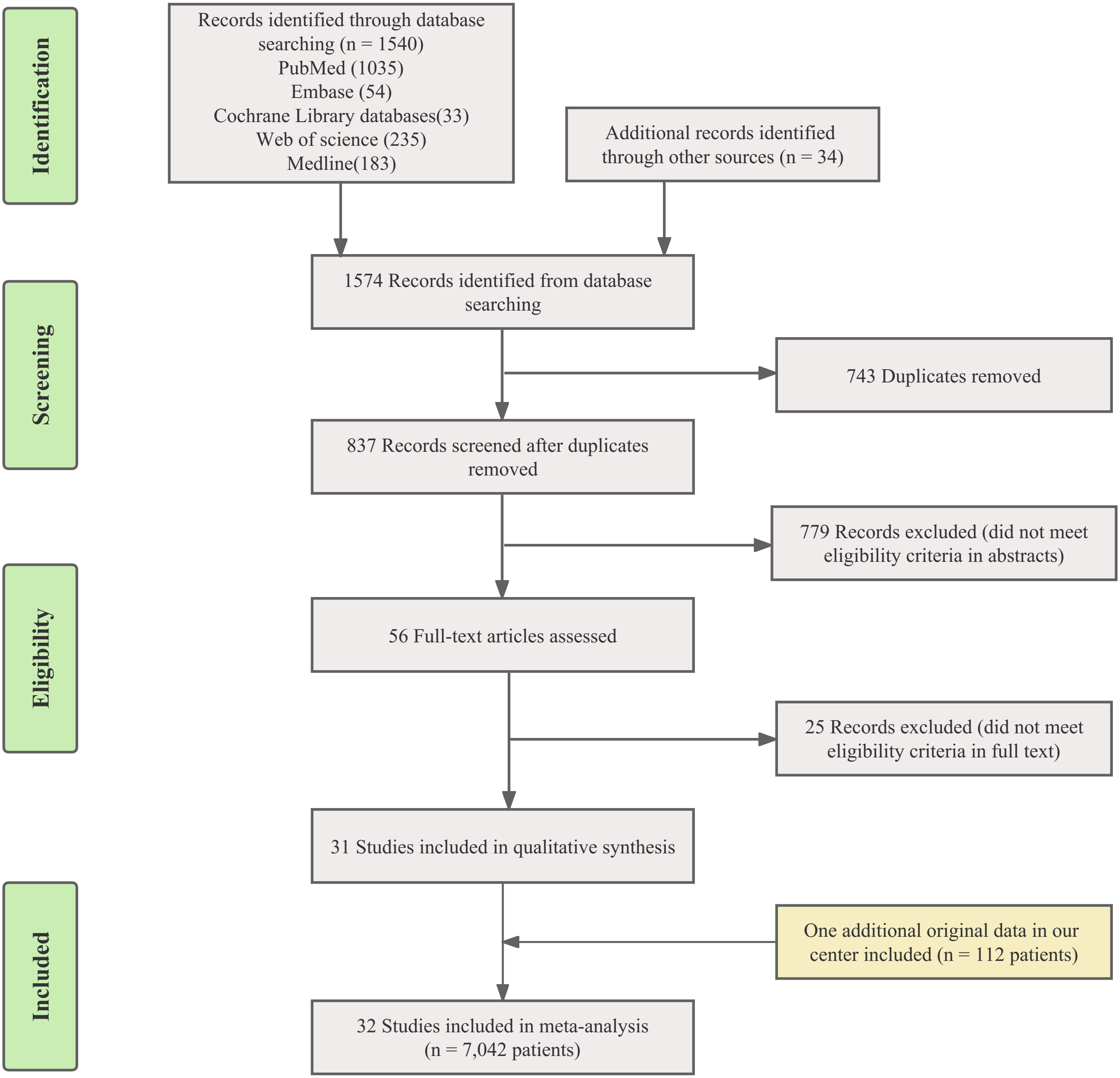

Supplementary Figure 2

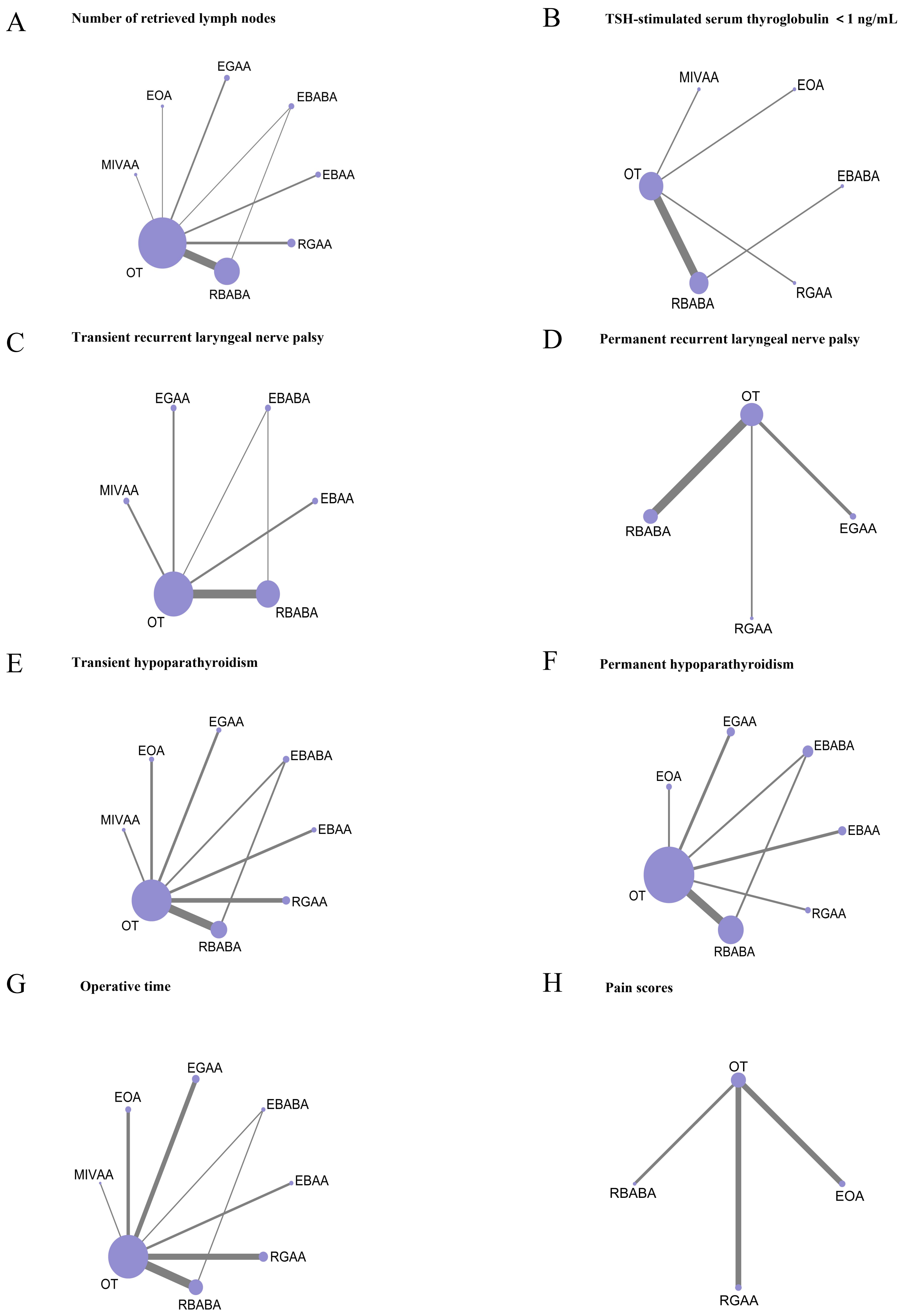

Supplementary Figure 3

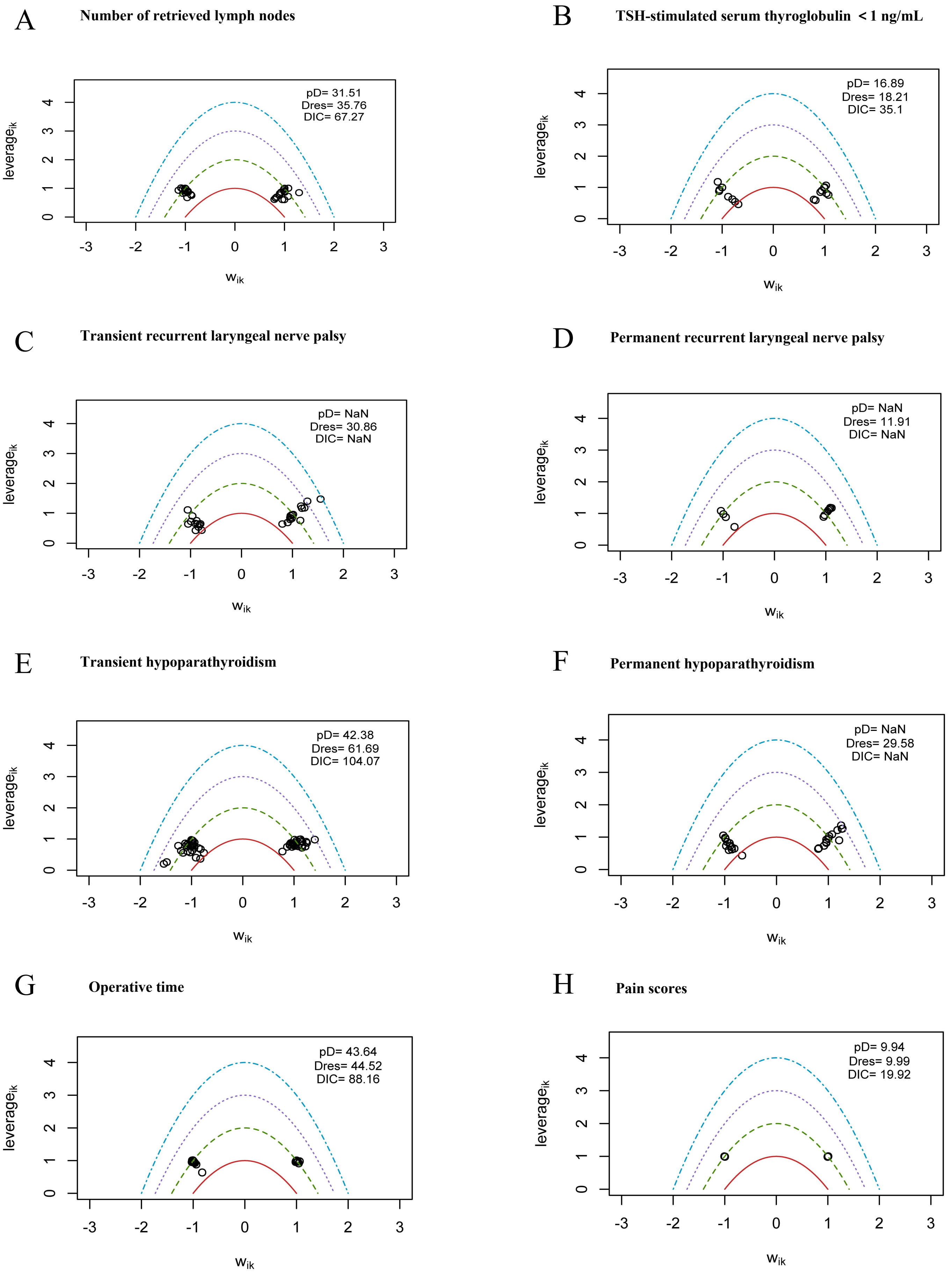

Supplementary Figure 4

A **Number of retrieved lymph nodes**

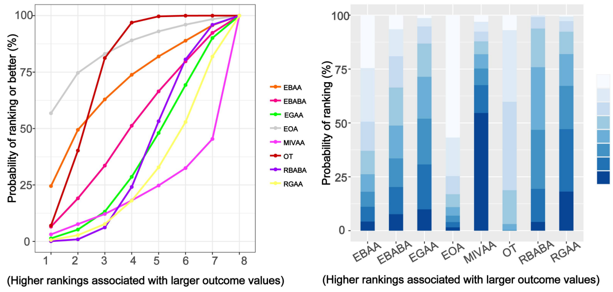

B **TSH-stimulated serum thyroglobulin ＜1 ng/mL**

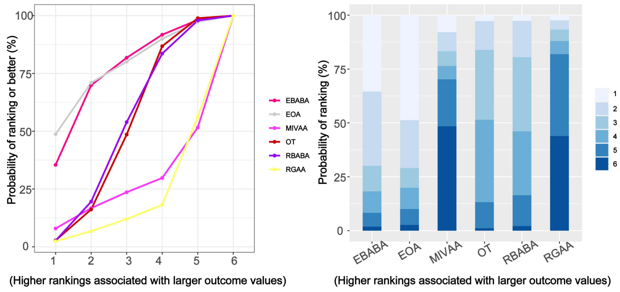

C **Transient recurrent laryngeal nerve palsy**

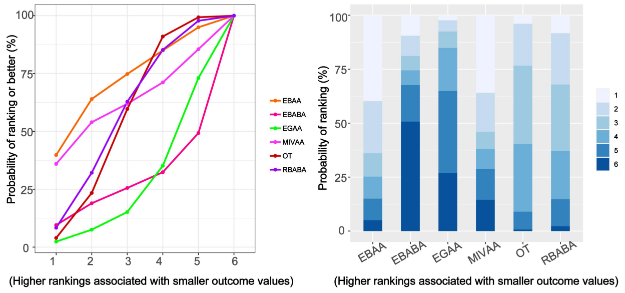

D **Permanent recurrent laryngeal nerve palsy**

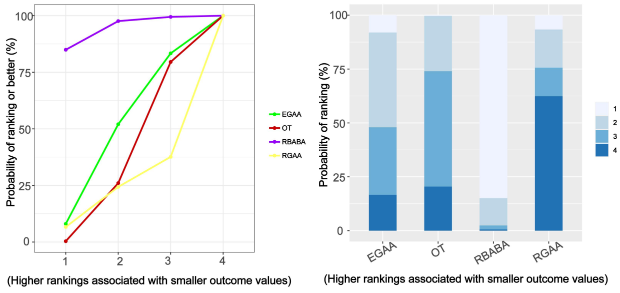

E **Transient hypoparathyroidism**

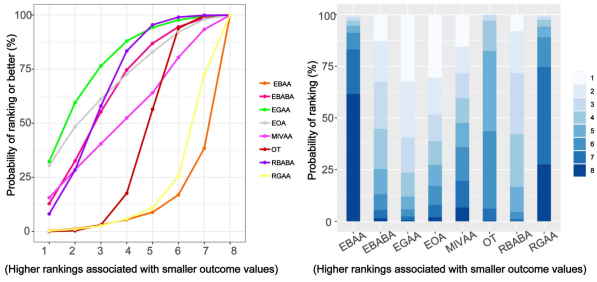

F **Permanent hypoparathyroidism**

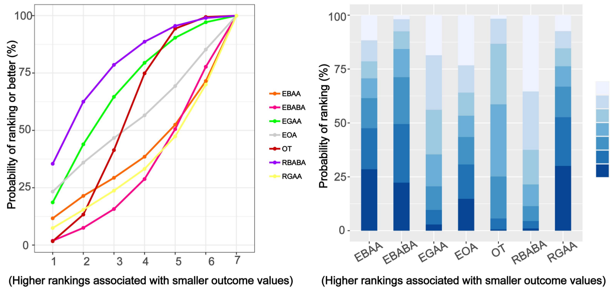

G **Operative time**

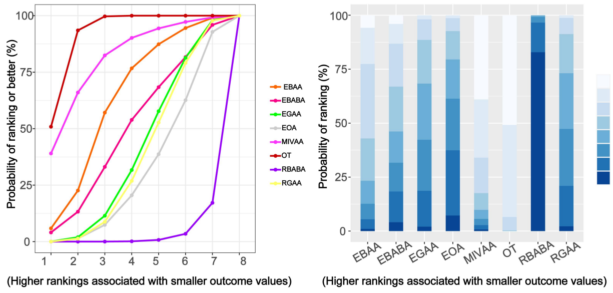

H **Pain scores**

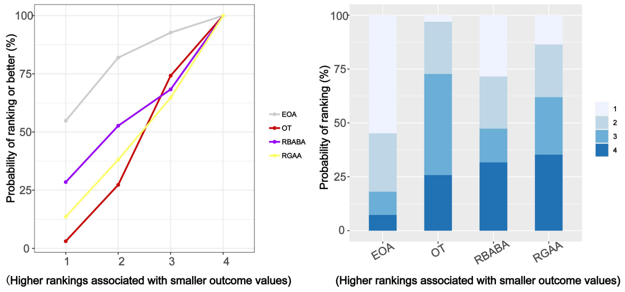

Supplementary Figure 5

A **Number of retrieved lymph nodes**

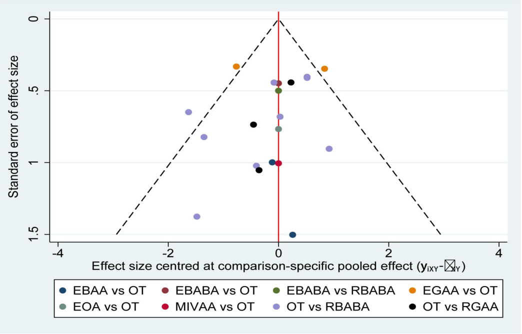

B **TSH-stimulated serum thyroglobulin ＜1 ng/mL**

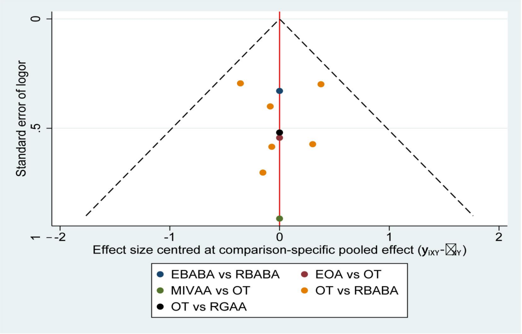

C **Transient recurrent laryngeal nerve palsy**

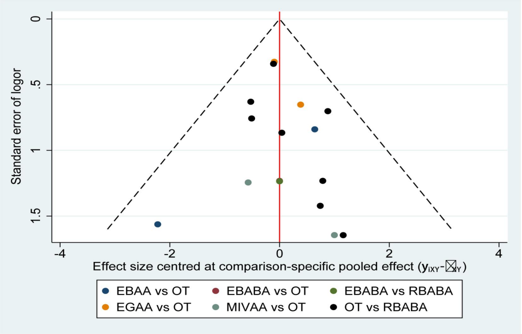

D **Permanent recurrent laryngeal nerve palsy**

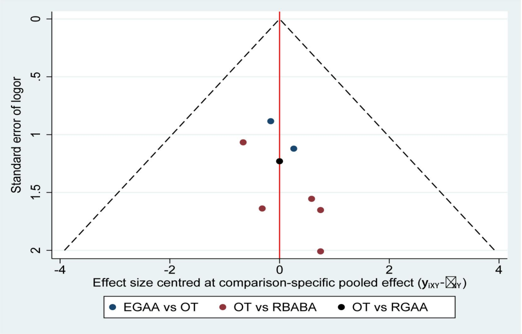

E **Transient hypoparathyroidism**

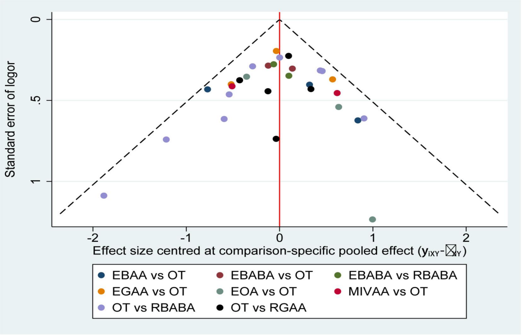

F **Permanent hypoparathyroidism**

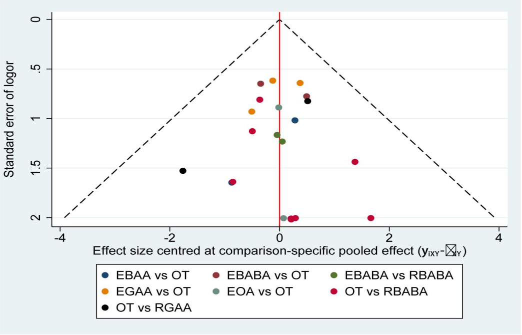

G **Operative time**

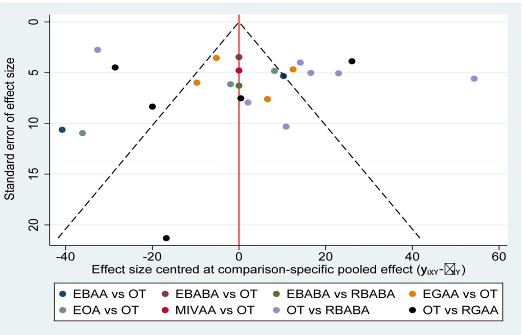

H **Pain scores**

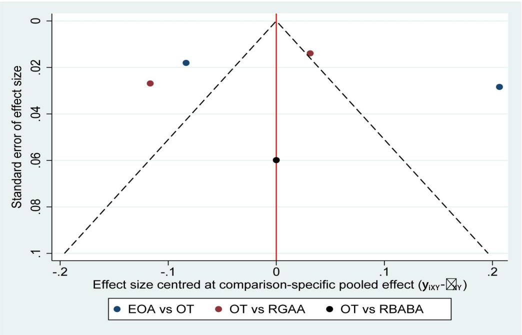

Supplementary Figure 6

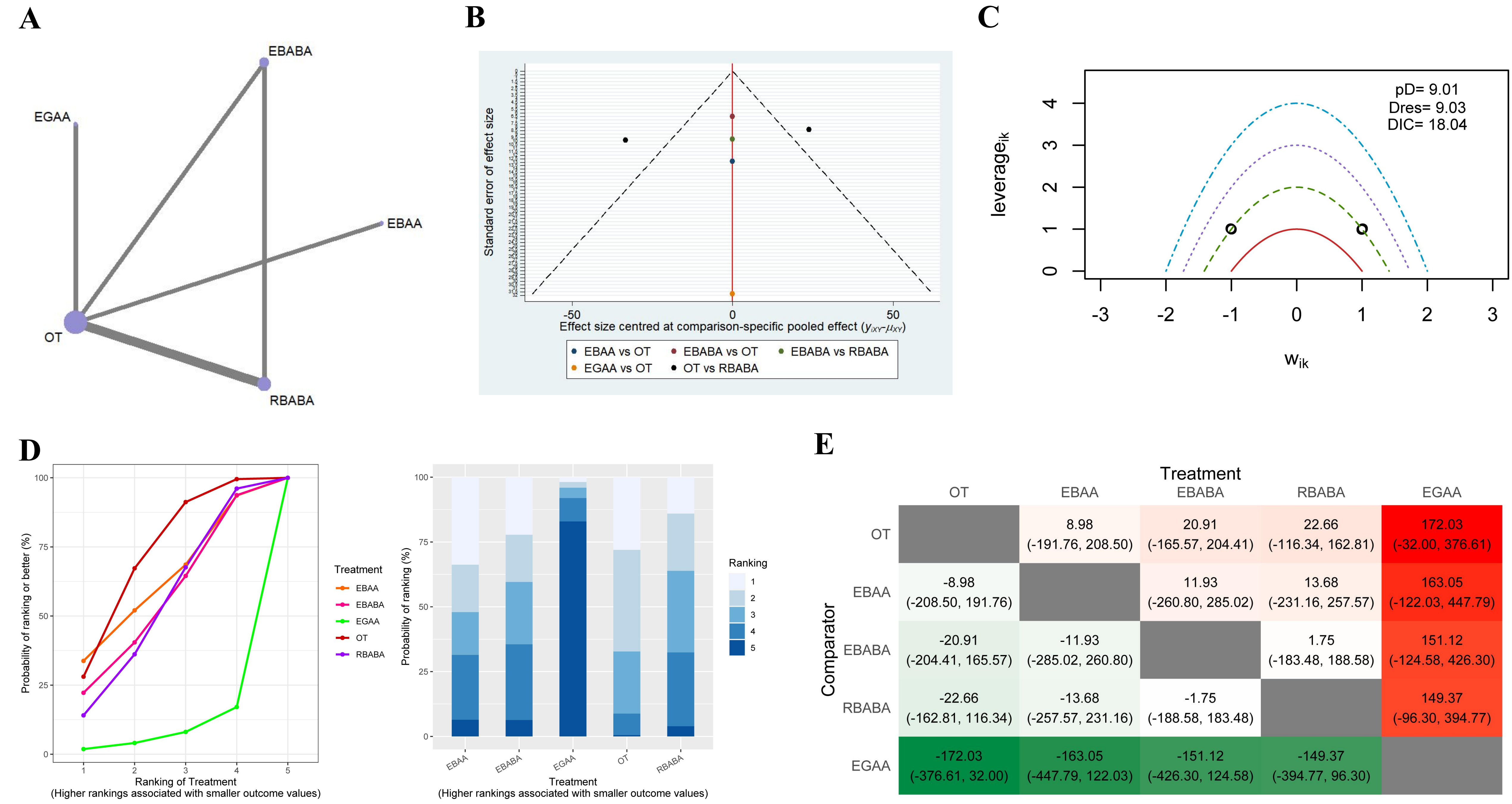

Supplementary Figure 7

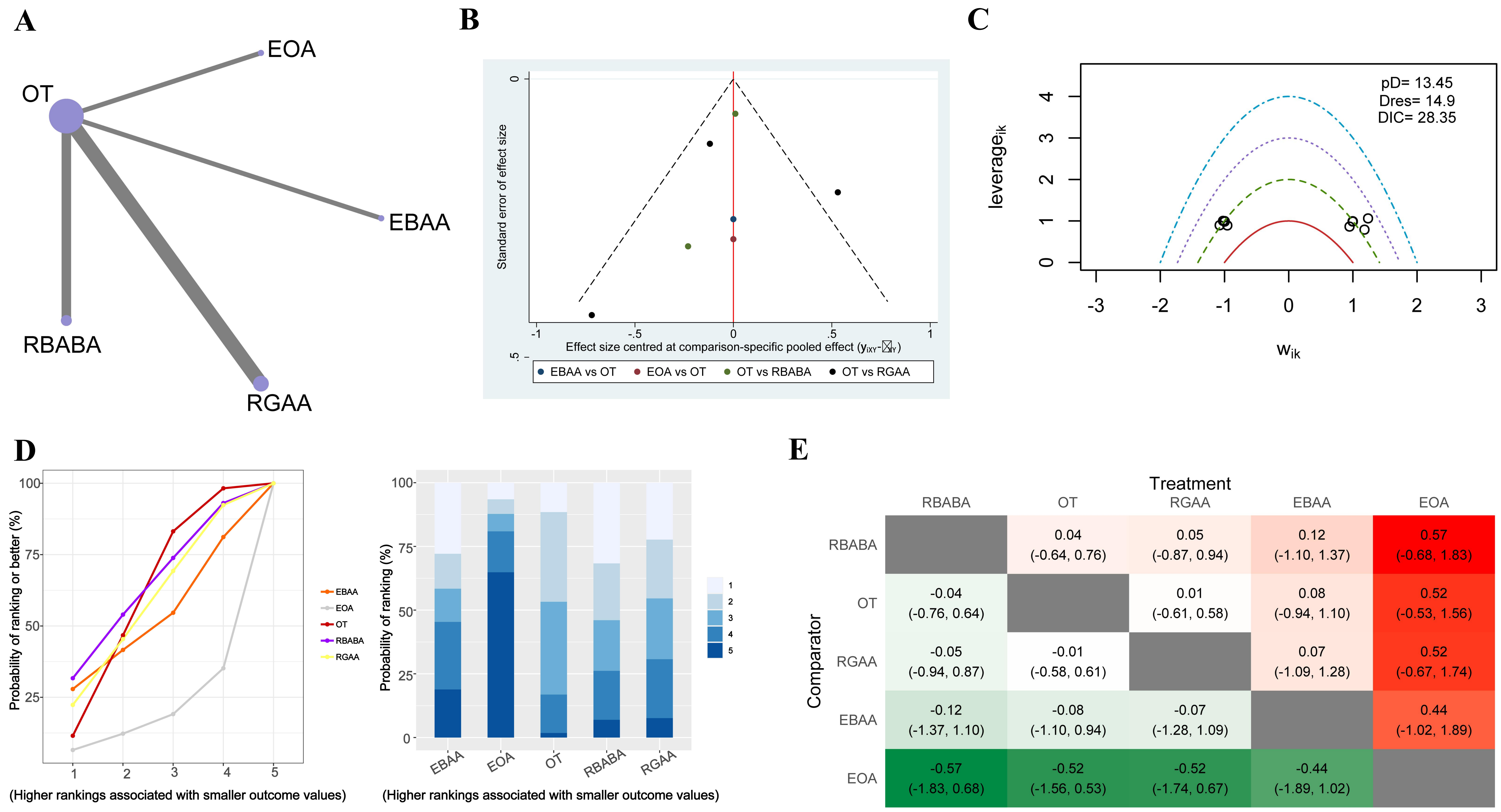

Supplementary Figure 8

```
. metabias _y _stderr, egger

Note: data input format theta se_theta assumed

Egger's test for small-study effects:
Regress standard normal deviate of intervention
  effect estimate against its standard error

.
Number of studies =   24                                  Root MSE      =    10.92
------------------------------------------------------------------------------
     Std_Eff |      Coef.   Std. Err.      t    P>|t|     [95% Conf. Interval]
-------------+----------------------------------------------------------------
       slope |   10.38509   31.02979     0.33   0.741    -53.96675    74.73694
        bias |   .8474342   6.096385     0.14   0.891    -11.79569    13.49056
------------------------------------------------------------------------------

Test of H0: no small-study effects          P = 0.891
```

Supplementary Table 1. The characteristics and surgical outcomes of patients in our center

Indicators	EBAA group (n = 58)	OT group (n = 54)
Age, mean (SD), year	34.74 (8.45)	34.91 (8.45)
Sex (male/female)	3/55	7/47
Tumor size, mean (SD), mm	7.18 (3.22)	8.63 (5.02)
Operative time, mean (SD), min	138.93 (34.55)	138.00 (20.57)
Number of retrieved lymph nodes, mean (SD)	9.36 (5.64)	9.19 (4.92)
Transient RLN palsy	2/58	6/54
Permanent RLN palsy	0/58	0/54
Transient hypoparathyroidism	20/58	17/54
Permanent hypoparathyroidism	2/58	2/54

Abbreviations: EBAA, endoscopic bilateral areola approach; OT, open thyroidectomy; RLN, recurrent laryngeal nerve.

Supplementary Table 2. The quality of included study assessed by the Newcastle-Ottawa scale

Study	Selection				Comparability	Outcome			Quality score
	Representativeness of the exposed cohort	Selection of the non-exposed cohort	Ascertainment of exposure	Demonstration that outcome of interest was not present at start of study	Comparability of cohorts on the basis of the design or analysis	Assessment of outcome	Was follow-up long enough for outcomes to occur	Adequacy of follow up of cohorts	
Lee et al.		★	★	★	★★		★		6
Kim et al.	★	★		★	★★	★		★	7
Lee et al.	★	★		★	★★	★	★	★	8
Lee et al.		★	★	★	★	★	★	★	7
Lombardi et al.	★	★	★	★	★	★	★	★	8
Noureldine et al.	★		★	★	★★		★	★	7
Ryu et al.	★	★		★	★★	★	★	★	8
Yi et al.	★	★	★	★	★		★	★	7
Lee et al.		★		★	★★	★	★	★	7
Kim et al.	★	★	★		★	★		★	6
Lee et al.	★	★	★		★★	★		★	7
Chai et al.	★	★	★	★	★★		★	★	8
He et al.	★		★	★	★★	★	★	★	8
Hensler et al.	★		★	★	★★		★	★	7
Huang et al.	★	★	★	★	★★	★	★		8

Kim et al.	★	★		★	★★		★	★	7
Kim et al.		★	★	★	★★	★	★	★	8
Koh et al.	★		★		★★	★		★	6
Lee et al.	★	★		★	★★	★	★	★	8
Park et al.	★	★		★	★★	★		★	7
Xiang et al.	★	★		★	★★		★	★	7
Chai et al.	★	★	★	★	★	★	★	★	8
Kim et al.	★		★		★★	★	★	★	7
Kim et al.	★		★	★	★★		★	★	7
Anuwong et al.	★	★	★	★	★★	★	★	★	9
Paek et al.	★		★	★	★★		★	★	7
Bae et al.	★	★	★	★	★	★	★	★	8
Zhang et al.	★	★		★	★★		★	★	7
Ahn et al.	★	★		★	★		★	★	6
Hong et al.	★		★	★	★★		★	★	7
Paek et al.	★		★	★	★	★	★	★	7
Yuan et al.	★	★	★	★	★	★	★	★	8

Supplementary Table 3. The surface under the cumulative ranking curve values of all outcomes

Group	Pain score	Operative time	Permanent hypoparathyroidism	Transient hypoparathyroidism	Permanent RLN palsy	Transient RLN palsy	STG < 1.0 ng/mL	Number of retrieved lymph nodes
EBAA	0.00	0.63	0.37	0.11	0.00	0.72	0.00	0.68
EBABA	0.00	0.50	0.30	0.65	0.00	0.27	0.75	0.50
EGAA	0.00	0.40	0.66	0.78	0.48	0.27	0.00	0.37
EOA	0.77	0.32	0.53	0.69	0.00	0.00	0.77	0.84
MIVAA	0.00	0.81	0.00	0.54	0.00	0.62	0.26	0.21
OT	0.35	0.92	0.54	0.39	0.35	0.55	0.51	0.75
RBABA	0.50	0.03	0.77	0.67	0.94	0.57	0.52	0.37
RGAA	0.39	0.38	0.33	0.17	0.23	0.00	0.19	0.28

Abbreviations: EBAA, endoscopic bilateral areola approach; EBABA, endoscopic bilateral axillo-breast approach; EGAA, endoscopic gasless transaxillary approach; EOA, endoscopic transoral approach; MIVAA, minimally invasive video-assisted approach; OT, open thyroidectomy; RBABA, robotic bilateral axillo-breast approach; RGAA, robotic gasless transaxillary approach; SUCRA, surface under the cumulative ranking curve; STG, TSH-stimulated serum thyroglobulin; RLN, recurrent laryngeal nerve.

Supplementary Table 4. Loop inconsistency and heterogeneity

Outcome of Interest	No. of Studies	No. of patients	Inconsistency		P Value	Heterogeneity, τ
			Loop	IF or RoR (95% CI)		
No. of retrieved LNs	18	3801	A-B-C	0.81(0.00-3.00)	0.47	0.35
Transient RLN palsy	14	2913	A-B-C	2.15 (1.00-67.70)	0.67	< 0.10
Transient hypoparathyroidism	27	6087	A-B-C	1.87 (1.00-4.69)	0.18	< 0.10
Permanent hypoparathyroidism	20	5509	A-B-C	1.08 (1.00-9.72)	0.95	< 0.10
Operative time	22	4261	A-B-C	48.78 (0.00-131.35)	0.25	>1.00

Abbreviations: IF, absolute difference between direct and indirect estimates; RoR, logarithm of the ratio of 2 odds ratios; LNs, lymph nodes; STG, TSH-stimulated serum thyroglobulin; RLN, recurrent laryngeal nerve. A: endoscopic bilateral axillary breast approach (EBABA); B, open thyroidectomy (OT); C: robotic bilateral axillary breast approach (RBABA).

Supplementary Table 5. Meta-regression of confounding covariates influencing heterogeneity

Covariates	Operative time (β, p value)
	A *vs*. B
Publication Years (After 2017 / before 2017)	0.20, 0.751
Publication types (matched / non-matched)	-0.71, 0.232
Publication types (retrospective / prospective)	-0.57, 0.394
Countries (Korea / non-Korea)	-0.55, 0.528

Abbreviations: β, regression coefficient. A, robotic bilateral axillo-breast approach (RBABA); B, open thyroidectomy (OT).
